
JOURNAL INFORMATION
==============================
NLM Title Abbreviation: Atlanta J Rec Med
NLM Journal ID: 0001011

Atlanta Journal-Record of Medicine

Publisher: Biomedical Journal Digitization Project

ARTICLE INFORMATION
==============================
PMCID: PMC9068464
Article version: 1
Subjects: Book Reviews

Book Reviews

Print publication date: 1912 Oct
Volume: 59
Issue: 7
First page: 405
Last page: 406
License: This work is free of known copyright restrictions. For more information, please see PMC Back Issue Digitization.
License URL: https://creativecommons.org/publicdomain/mark/1.0/

==============================

Professionally *tested*—Approved professionally.
**Exceptionally Palatable, Digestible, Dependable**
Physicians have been able to prescribe to advantage
### HYDROLEINE
in cases in which cod-liver oil is indicated. Hydroleine is the pure Norwegian cod-liver oil emulsified in a manner which makes it extremely utilizable. It is without medicinal admixture.
*Sold by Druggists*
THE CHARLES N. CRITTENTON CO., 115 FULTON ST., NEW YORK
Sample with literature sent to physicians on request to explain their extraordinary influence, at least when followed by ether, over the entire nervous system? Is there merely an association of ideas? The smell of ether is associated with the surgical knife in the minds of adults, and is terrifying to children from its irritating qualities. Perhaps the very familiarity and the harmlessness associated with the odors of flowers and fruit are sufficent to suggest powerfully to the subject that what he is about to undergo cannot be dangerous or even unfamiliar.

### BOOK REVIEWS

PSYCHOTHEROPY. By James J. Walsh, M.D., Ph.D., Dean and Prof. of Functional Diseases and the History of Medicine at Fordham University School of Medicine. 806 pages. Published by D. Appleton & Co., New York.

This work includes the history of the Use of Mental Influence directly and indirectly, in the principles for application of energies derived from the mind to the treatment of disease.

It is one of the most thorough and systematic books yet published on Psychotheropy.

MODERN METHODS IN NURSING. By Georgiana J. Sanders, Formerly Superintendent of Nurses at the Massachusetts General Hospital, Boston. 12mo of 881 pages, with 228 illustrations. Cloth, $2.50 net. Published by W. B. Saunders Co., 1912. Philadelphia and London.

This book is what Medicine and Surgery have adopted to the requirements of the trained nurse.

---

DUODENAL ULCER. By B. G. A. Moynihan, M.D., (Lond.) T. R. C. S. Leads. Second edition, enlarged, illustrated. 486 pages. Published by W. B. Saunders Co., Philadelphia

This valuable and most interesting work has been changed chiefly in this edition, in the portion concerned with the differential diagnosis of duodenal ulcer and the X-ray examinations of the stomach after the administration of bismuth.

---

ELEMENTARY BACTERIOLOGICAL AND PROTOZOOLOGY: THE MICROBIOLOGICAL CAUSES OF THE INFECTIOUS DISEASES. By Herbert Fox, M.D., Director of the William Pepper Laboratory of Clinical Medicine in the University of Pennsylvania. 12mo, 237 pages, with 67 engravings and 5 colored plates. Cloth, $1.75, net. Lea & Febiger, Philadelphia and New York, 1912.

This work is designed as an elementary text-book of Bacteriology and Protozoology for nurses and beginners. Without being technical, it gives a good idea of the nature of micro-organisms, and then discusses with more emphasis the ways in which bacteria pass from one individual to another, how they enter the body and act when once within, and their manner of exit. Such general information concerning the character of the disease process has been included as seemed necessary to clarify the nature of microbe action.
